# Primary ocular-adnexal non-Hodgkin Lymphoma in a Peruvian cohort: An 18-year experience

**DOI:** 10.1371/journal.pone.0356381

**Published:** 2026-08-25

**Authors:** Renato Luque-Benavides, Gabriel De la Cruz Ku, Bryan Valcarcel, Alejandro Garcia, Fiorella Davila Galecio, Alexandra Luque, Maria Guadalupe Lujan Peche, Deiby Cruzado Sanchez, Daniel Enriquez-Vera

**Affiliations:** 1 Universidad Científica del Sur, Lima, Perú; 2 The George Washington University, Washington DC, United States of America; 3 Temple University Hospital, Philadelphia, Pennsylvania, United States of America; 4 Department of Ophthalmology, Instituto Nacional de Enfermedades Neoplásicas, Lima, Peru; 5 Department of Hematology and Medical Oncology, Instituto Nacional de Enfermedades Neoplásicas, Lima, Peru; 6 Escuela Profesional de Medicina Humana, Universidad Peruana San Juan Bautista, Lima, Peru; European Institute of Oncology, ITALY

## Abstract

**Background:**

Primary ocular-adnexal non-Hodgkin lymphoma (POANHL) is a rare malignancy, but the most common tumor of the ocular adnexa. Data on real-world outcomes in Hispanic-Latino populations are limited, despite potential geographic and biologic differences. We describe the treatment and outcomes of POANHL in a Peruvian cohort and identify factors associated with survival outcomes.

**Methods:**

We conducted a retrospective cohort study including all patients diagnosed with localized POANHL (stage I and II) at a tertiary referral center between 2000 and 2017. Survival outcomes were estimated using Kaplan–Meier methods, and Cox proportional hazards models were used to identify prognostic factors for event-free survival (EFS) and overall survival (OS).

**Results:**

A total of 98 patients were included. The mean age was 58.0 ± 17.5 years. The orbit was the most frequently involved site (42.8%), followed by the eyelid (29.6%) and the conjunctiva (27.6%). The most common associated lymphomas were extranodal marginal zone lymphoma (50.0%), diffuse large B-cell lymphoma (23.5%), and follicular lymphoma (7.1%). Most patients presented with stage I disease (80.6%), good performance status (ECOG 0–1: 88.8%), and low international prognostic index (0–1: 69.6%). Treatment included chemotherapy (39.2%), radiotherapy (24.5%), combined modality therapy (12.2%), and observation/no treatment (35.7%). Cyclophosphamide, vincristine, doxorubicin, and prednisone-based regimens were the most commonly used chemotherapy backbone. Complete response (CR) was 45.6% among treated patients. With a median follow-up of 20.2 years, 5-year OS and EFS were 94.8% and 79.2% for indolent lymphomas, and 59.7% and 42.8% for aggressive lymphomas respectively. On multivariable analysis, older age was independently associated with worse outcomes in indolent lymphoma, both for EFS (HR = 1.084, 95%CI 1.028–1.142) and OS (HR = 1.092, 95%CI: 1.037–1.150). In aggressive lymphoma, treatment modality was the only independent prognostic factor for DFS, with chemotherapy (HR 0.135, 95% CI 0.031–0.595; p = 0.008) and chemoradiotherapy (HR 0.112, 95% CI 0.023–0.547; p = 0.007) associated with improved outcomes vs. observation; radiotherapy showed a non-significant trend (HR = 0.200, 95%:CI 0.033–1.203). No variables were independently associated with OS in aggressive lymphoma.

**Conclusions:**

We report real-world outcomes of POANHL in a Hispanic–Latino cohort, demonstrating distinct clinical characteristics and survival outcomes compared with previously published series.

## Introduction

Primary ocular-adnexal non-Hodgkin lymphoma (POANHL) is a rare extranodal lymphoma for which high quality evidence on real-world treatment outcomes remains limited, particularly in underrepresented populations such as those from Latin America [1,2]. Most available evidence consists of single-institution retrospective series, and published cohorts are generally small despite considerable heterogeneity in histologic subtype, treatment strategy, and long-term outcomes. As a result, important questions regarding prognostic factors and real-world treatment effectiveness remain incompletely characterized. Geographic differences in disease distribution, histologic subtype, and potential etiologic factors further emphasize the importance of reporting outcomes from diverse populations, including regions that remain underrepresented in the current literature, as findings from other regions may not be fully generalizable [3–7]. This gap is clinically relevant, as evaluating real-world outcomes is critical to assess treatment effectiveness and generate evidence that can inform therapeutic strategies in routine practice.

POANHL accounts for approximately 2% of all non-Hodgkin lymphomas but represents the most common malignancy of the ocular adnexal region [1,8]. This pathology is predominantly indolent, with extranodal marginal zone lymphoma of mucosa-associated lymphoid tissue (MALT) as the most frequent subtype, generally associated with favorable survival outcomes [1,3,9–11]. However, prognosis varies according to histologic subtype and disease extent, with aggressive entities such as diffuse large B-cell lymphoma demonstrating inferior survival [12,13]. Additional prognostic factors consistently associated with inferior survival include advanced Ann Arbor stage, older age, poor performance status, and bulky local disease [7,14,15]. Despite radiotherapy being widely used as first-line treatment for localized disease, there is no universally accepted standard of care, and substantial variability persists in treatment approaches [14,16,17].

In this context, we present an 18-year experience from Peru, representing one of the largest reported single-center POANHL cohorts from Latin America. Our study aimed to describe real-world outcomes of POANHL, evaluate associated prognostic factors, and contextualize our findings within existing global literature. Beyond expanding the available literature from an underrepresented region, this study contributes additional long-term real-world evidence regarding treatment patterns, prognostic factors, and survival outcomes for this uncommon malignancy.

## Methods

### Population

We performed a retrospective cohort study and reviewed the medical records of patients diagnosed with POANHL between January 2000 and December 2017 at the Instituto Nacional de Enfermedades Neoplásicas (INEN), Lima, Peru. The patients were followed until December 2025. Data was accessed from July 15^th^, 2019 to December 10^th^, 2022. Instituto Nacional de Enfermedades Neoplásicas (INEN) is the national tertiary referral center for oncology in Peru and serves as the country’s principal referral center for hematologic malignancies. Patients were referred from throughout Peru for diagnosis and management [18]. Patients were eligible for inclusion if they had a histopathologically and immunophenotypically confirmed diagnosis of primary ocular adnexal non-Hodgkin lymphoma, defined as lymphoma arising within the ocular adnexal tissues (orbit, conjunctiva, eyelid, or lacrimal gland) at presentation, without evidence of disseminated (Ann Arbor stage III–IV) disease. Eligible patients were identified using institutional databases from the Departments of Ophthalmology and Pathology.

### Treatment exposure

Therapeutic management was classified into four categories: radiotherapy (RT), chemotherapy (CT), combined-modality chemoradiotherapy (CRT), or observation/no treatment [19]. Treatment strategies were guided by the Ann Arbor stage and the extent and anatomical location of ocular adnexal involvement [20]. Treatment strategies were guided by the Ann Arbor stage, histologic subtype, and extent of ocular adnexal involvement. Patients with early-stage disease (Ann Arbor I–II) were generally managed with radiotherapy, chemotherapy, combined chemoradiotherapy, or observation according to histologic subtype and clinical characteristics. For orbital tumors, RT was commonly delivered to the entire orbit using a wedge-pair technique with inclusion of the orbital cone, although a subset of patients received intensity-modulated RT (IMRT). Radiation dose was determined by the treating radiation oncologist and ranged from 20 to 50 Gy, most commonly 36 Gy administered in 20–25 fractions.

### Study variables

The following baseline variables recorded at the time of diagnosis, were collected from medical records: sex (female/male), age (years), presenting symptoms, Eastern Cooperative Oncology Group (ECOG) performance status, tumor size, histologic grade (indolent versus aggressive/highly aggressive), histological subtype, anatomical location within the ocular-adnexal region (OAR), bilaterality, and Ann Arbor cancer stage [20]. Primary OAR disease sites included the orbit, conjunctiva, lacrimal gland, and eyelids, and were classified based on ophthalmologic examination and radiological imaging.

The International Prognostic Index (IPI) was calculated for each patient using the conventional IPI criteria available at the time of diagnosis. Given the inclusion of multiple B-cell and T-cell non-Hodgkin lymphoma subtypes over an 18-year study period, a standardized IPI scoring system was applied uniformly across the entire cohort, without modification according to histologic subtype, to facilitate comparison of prognostic risk between patients.

Treatment response was assessed using the Lugano Classification [20], adapted for ocular adnexal involvement. Complete response (CR) was defined as disappearance of all detectable disease, partial response (PR) as ≥50% reduction, stable disease (SD) as neither sufficient shrinkage nor progression, and progressive disease (PD) as ≥25% increase in lesion size or new lesions. Assessments were based on clinical ophthalmologic examination, imaging (CT), and slit-lamp evaluation, performed 4–12 weeks after therapy and every 3–6 months during the first 2 years of follow-up.

OS and event-free survival (EFS) were the primary outcomes of interest. OS was defined as the interval from diagnosis to death from any cause or last follow-up [21]. EFS was defined as the time from diagnosis to the first documented event, including disease relapse, disease progression, initiation of second-line therapy, or death [21]. To ascertain vital status, patients’ National Identification Document numbers were linked with the National Registry of Identification and Civil Status of Peru.

### Statistical analysis

Descriptive statistics were used to assess sociodemographic, clinical, pathological, surgical, and therapeutic characteristics. We used mean and standard deviation for quantitative variables; frequencies and percentages were used to assess qualitative variables. Comparisons of clinical and sociodemographic characteristics were performed using the Chi-square test or Fisher’s exact test when appropriate. Follow-up time was calculated from the date of diagnosis to the date of last contact or death. Patients who were lost to follow-up were censored at the date of last contact. Median follow-up was estimated using the reverse Kaplan-Meier method. Survival probabilities were estimated using the Kaplan-Meier method, and group differences were estimated using the log-rank test. A two-sided p-value <0.05 was considered statistically significant. For OS and EFS, survival analyses were performed separately for patients with indolent and aggressive/highly aggressive lymphoma. Separate multivariable Cox proportional hazards regression models were constructed for indolent and aggressive/highly aggressive lymphomas, adjusting for age, sex, ECOG performance status, clinical stage, and treatment modality. To address any potential bias in our analyses, all population available was included in our analyses; no sample was calculated. All statistical analyses were performed using IBM SPSS Statistics for Windows, version 26 (IBM Corp., Armonk, NY, USA).

### Ethics

This study was approved by the Institutional Review Board (IRB) of the Instituto Nacional de Enfermedades Neoplásicas (INEN) (INEN 19–23). Due to the retrospective nature of this study, the consent was waived by the ethics committee. Data was collected confidentially by the investigators after obtaining authorization to access the medical records by the IRB at the institution. All data was handled with strict confidentiality and only used for study purposes; it was de-identified, and clinical information was codified S1 File.

## Results

### Sociodemographic and clinical characteristics

**Fig 1:**
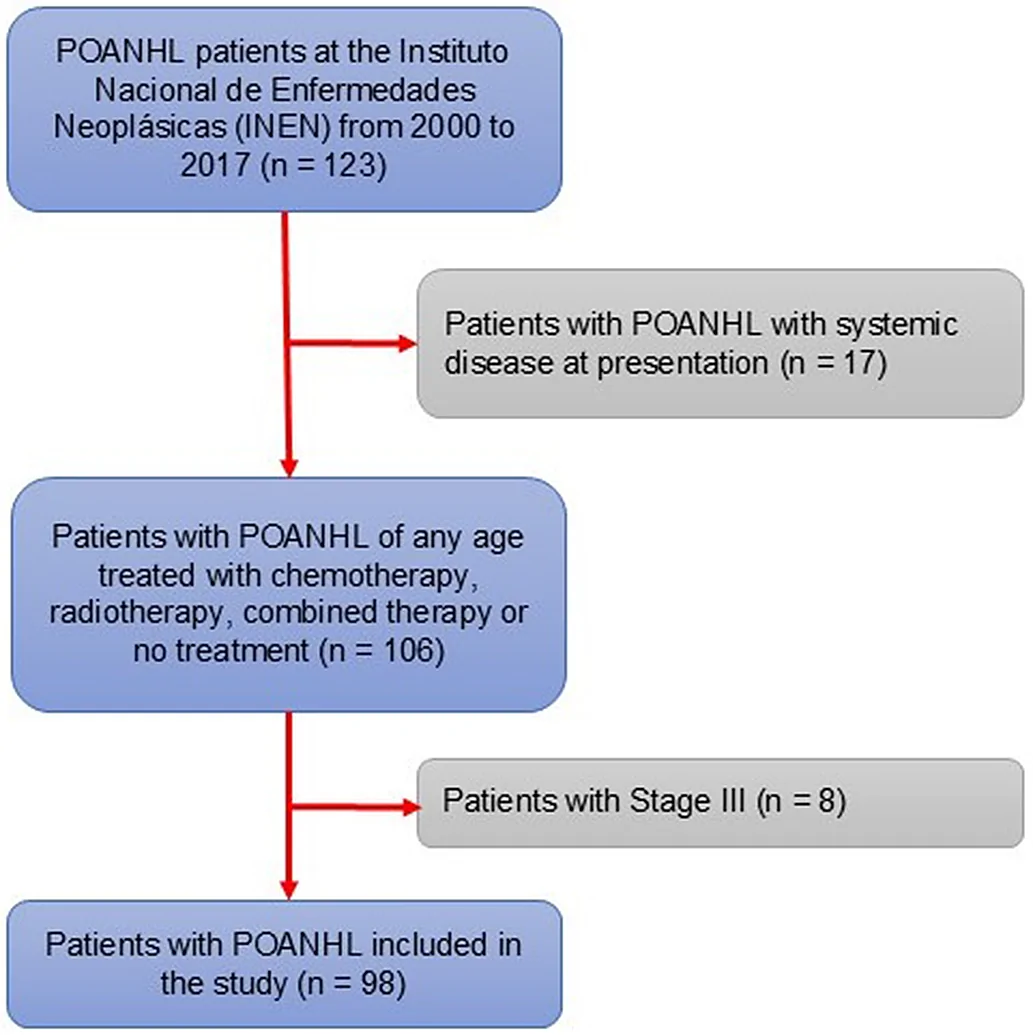
Flow chart of patients included in the study.

From a total of 123 patients with POANHL, 98 met the inclusion criteria (Fig 1). Indolent lymphomas accounted for 59.2% of cases, while aggressive/highly aggressive subtypes represented 40.8%. Among the 98 included patients, the most frequent histologic subtype was MALT lymphoma (49, 50.0%), followed by diffuse large B-cell lymphoma (DLBCL) (23, 23.5%), follicular lymphoma (7, 7.1%), mantle cell lymphoma (3, 3.1%), B-lymphoblastic lymphoma (4, 4.1%), and small lymphocytic lymphoma (1, 1.0%). Peripheral T-cell lymphomas accounted for 7 cases (7.1%), and T/NK-cell lymphomas for 4 cases (4.1%). B-cell lymphomas comprised 88.8% of cases, whereas T-cell and NK/T-cell lymphomas together accounted for 11.2% of cases.

The mean age at diagnosis was 58.0 ± 17.5 years, and 48.0% of patients were female. Overall, the most common site was the orbit (42.8%), followed by the eyelid (29.6%) and conjunctiva (27.6%). Eyelid involvement was more frequent among aggressive/highly aggressive lymphomas (45.0% vs 19.0%), whereas conjunctival disease was more common in indolent subtypes (31.5% vs 17.5%; p = 0.018) (Table 1).

**Table 1:** Sociodemographic and clinical characteristics of Hispanic patients with ocular-adnexal non-Hodgkin lymphoma stratified by tumor aggressiveness.

Characteristics	Indolent N = 58	Aggressive / Highly aggressive N = 40	Total N = 98	p-value
Age -mean (SD)	60.10 (14.79)	55.03 (20.70)	58.03 (17.53)	0.160 ^b^
Sex				
Female	23 (39.7)	24 (60.0)	47 (48.0)	0.048 ^a^
Male	35 (60.3)	16 (40.0)	51 (52.0)	
Length of disease – mean (SD)	9.55 (10.39)	7.00 (8.59)	8.51 (9.74)	0.204 ^b^
Proptosis	26 (44.8)	25 (62.5)	51 (52.0)	0.085 ^a^
Location				
Eyelid	11 (19.0)	18 (45.0)	29 (29.6)	0.018 ^a^
Conjunctiva	20 (31.5)	7 (17.5)	27 (27.6)	
Orbit	27 (46.6)	15 (37.5)	41 (42.8)	
Tumor size >2 cm	22 (45.8)	24 (77.4)	46 (58.2)	0.005 ^a^
Stage				
I	48 (82.8)	31 (77.5)	79 (80.6)	0.603 ^a^
II	10 (17.2)	9 (22.5)	19 (19.4)	
IPI				
0-1	33 (75.0)	22 (62.9)	55 (69.6)	0.842 ^a^
≥2	11 (25.0)	13 (37.1)	24 (30.4)	
Bilateral involvement	5 (8.1)	3 (7.5)	8 (8.2)	0.842 ^a^
ECOG				
0-1	55 (94.8)	32 (80.0)	87 (88.8)	0.022 ^a^
2-4	3 (5.2)	8 (20.0)	11 (11.2)	
B symptoms	2 (3.4)	5 (12.5)	7 (7.1)	0.087 ^a^
Treatment				
Antibiotics	4 (6.9)	1 (2.5)	5 (5.1)	0.331 ^a^
Surgery	4 (6.9)	3 (7.5)	7 (7.1)	0.909 ^a^
Chemotherapy	12 (20.7)	26 (66.7)	38 (39.2)	<0.001 ^a^
Radiotherapy	21 (36.2)	3 (7.5)	24 (24.5)	0.001 ^a^
Chemoradiotherapy	4 (6.9)	8 (20.0)	12 (12.2)	0.062 ^a^
None	24 (41.4)	11 (27.5)	35 (35.7)	<0.001 ^a^
Response to therapy				
Complete	21 (53.8)	10 (34.5)	31 (45.6)	0.129 ^a^
Partial	12 (30.8)	16 (38.5)	28 (41.2)	
Stable or Progression	6 (15.4)	3 (10.3)	9 (13.2)	

^a^Chi-square test

^b^Student t test

SD, standard deviation; IPI, International Prognostic Index; ECOG, Eastern Cooperative Oncology Group performance status

A tumor diameter >2 cm was identified in 58.2% of patients and occurred more frequently in aggressive/highly aggressive than in indolent lymphomas (77.4% vs 45.8%; p = 0.005). Most patients presented with early-stage disease, with 80.6% diagnosed at clinical stage I and 19.4% at stage II, with no significant differences between histologic subtypes (p = 0.603) (Table 1).

Regarding the International Prognostic Index (IPI), 69.6% of patients had low-risk scores (0–1), whereas 30.4% had scores ≥2, with no significant differences between histologic subtypes (p = 0.842). Bilateral involvement was observed in 8.2% of cases, without differences between subtypes (p = 0.842). Overall performance status was favorable, with 88.8% of patients presenting an ECOG score of 0–1. Poor performance status (ECOG 2–4) was significantly more frequent in aggressive/highly aggressive lymphomas than in indolent subtypes (20.0% vs 5.2%; p = 0.022). B symptoms were present in 7.1% of patients and were more common in aggressive/highly aggressive disease (12.5% vs 3.4%; p = 0.087), although this difference did not reach statistical significance (Table 1).

### Treatment patterns

Treatment patterns varied according to disease stage and histologic subtype. Among patients with localized stage I disease, radiotherapy alone was the predominant treatment modality in indolent lymphomas (39.6%), whereas aggressive histologies were more frequently managed with systemic chemotherapy or combined chemoradiotherapy. Observation without immediate treatment occurred primarily among patients with indolent disease (43.8%).

Among patients with stage II disease, systemic therapy became more common regardless of histology (indolent, 30.0%; aggressive, 45.0%). Among patients receiving chemotherapy, CHOP-based regimens constituted the most common chemotherapy backbone (72.7%), whereas radiotherapy remained an important component of treatment for selected localized presentations (7.5%).

Overall, 5.1% of patients received antibiotics, 7.1% underwent surgery, 39.2% received chemotherapy, 24.5% received radiotherapy alone, and 12.2% received combined chemoradiotherapy. No local or systemic therapy was administered to 35.7% of patients, more frequently among those with indolent than aggressive/highly aggressive lymphomas (41.4% vs. 27.5%; p < 0.001). Chemotherapy was significantly more common in aggressive/highly aggressive disease (66.7% vs. 20.7%; p < 0.001), whereas radiotherapy alone was more frequently used in indolent disease (36.2% vs. 7.5%; p = 0.001). Surgical and antibiotic-based treatments were uncommon in both groups (Table 1).

### Treatment response and toxicity

Treatment response differed according to histologic subtype. Among patients with indolent lymphoma, complete response was achieved in 53.8% of treated patients, partial response in 30.8%, and stable or progressive disease in 15.4%. Patients with aggressive/highly aggressive lymphoma demonstrated lower complete response rates (34.5%) and higher partial response rates (38.5%), while stable or progressive disease occurred in 10.3%. However, these differences did not reach statistical significance (p = 0.129) (Table 1).

Among patients who received CHOP-based chemotherapy, complete response was achieved in 39.1% of cases. Overall, complete response was observed in 45.6% of treated patients, while partial response occurred in 41.2% and stable or progressive disease in 13.2%.

Treatment-related adverse events documented in the medical record were uncommon and were predominantly mild. Conjunctivitis was the most frequently reported toxicity (4.1%), followed by cataracts and keratitis (2.0% each). Because adverse events were collected retrospectively from medical records, the incidence of treatment-related toxicity may be underestimated and should be interpreted cautiously.

### Survival

Survival outcomes differed according to histologic subtype; therefore, analyses are presented stratified by lymphoma aggressiveness.

**Fig 2:**
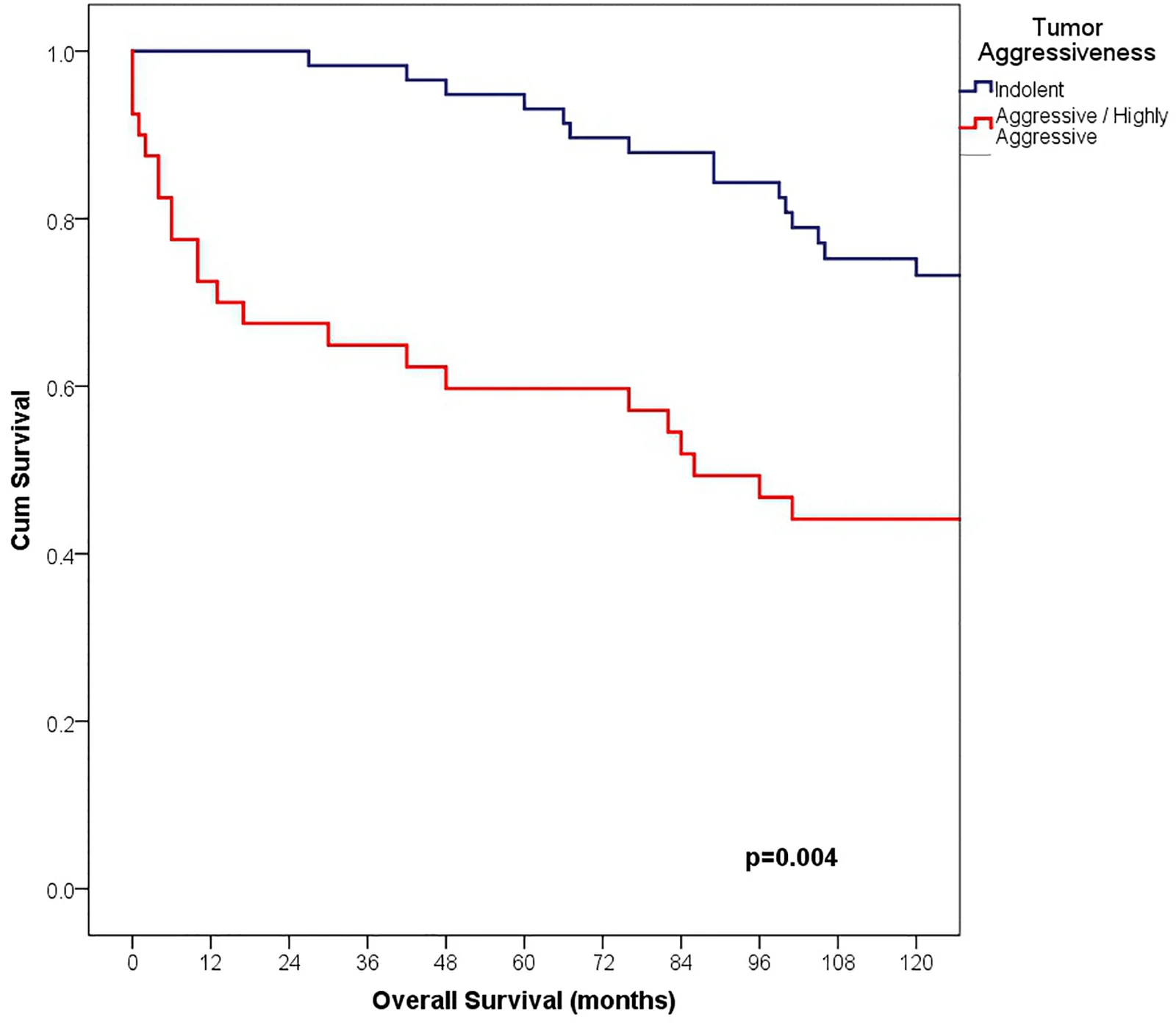
Kaplan–Meier overall survival curve of Hispanic patients with ocular-adnexal non-Hodgkin lymphoma stratified by tumor aggressiveness.

**Fig 3:**
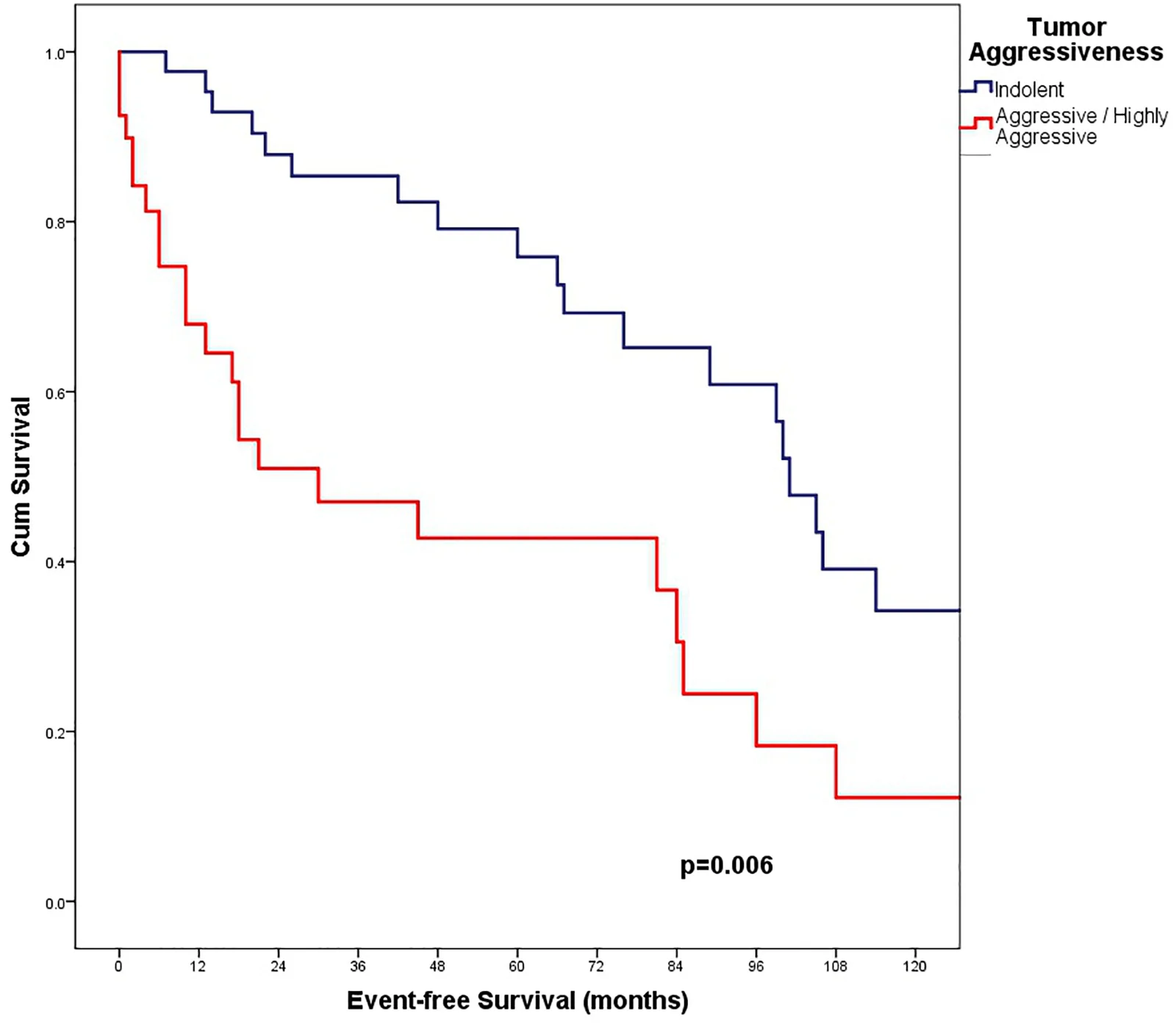
Kaplan–Meier event-free survival curve of Hispanic patients with ocular-adnexal non-Hodgkin lymphoma stratified by tumor aggressiveness.

Patients with indolent lymphoma demonstrated significantly more favorable outcomes than those with aggressive lymphoma. Five-year overall survival was 94.8% among patients with indolent disease compared with 59.7% among patients with aggressive disease. Similarly, five-year event-free survival was 79.2% and 42.8%, respectively (both p < 0.01) (Figs 2 and 3).

**Fig 4:**
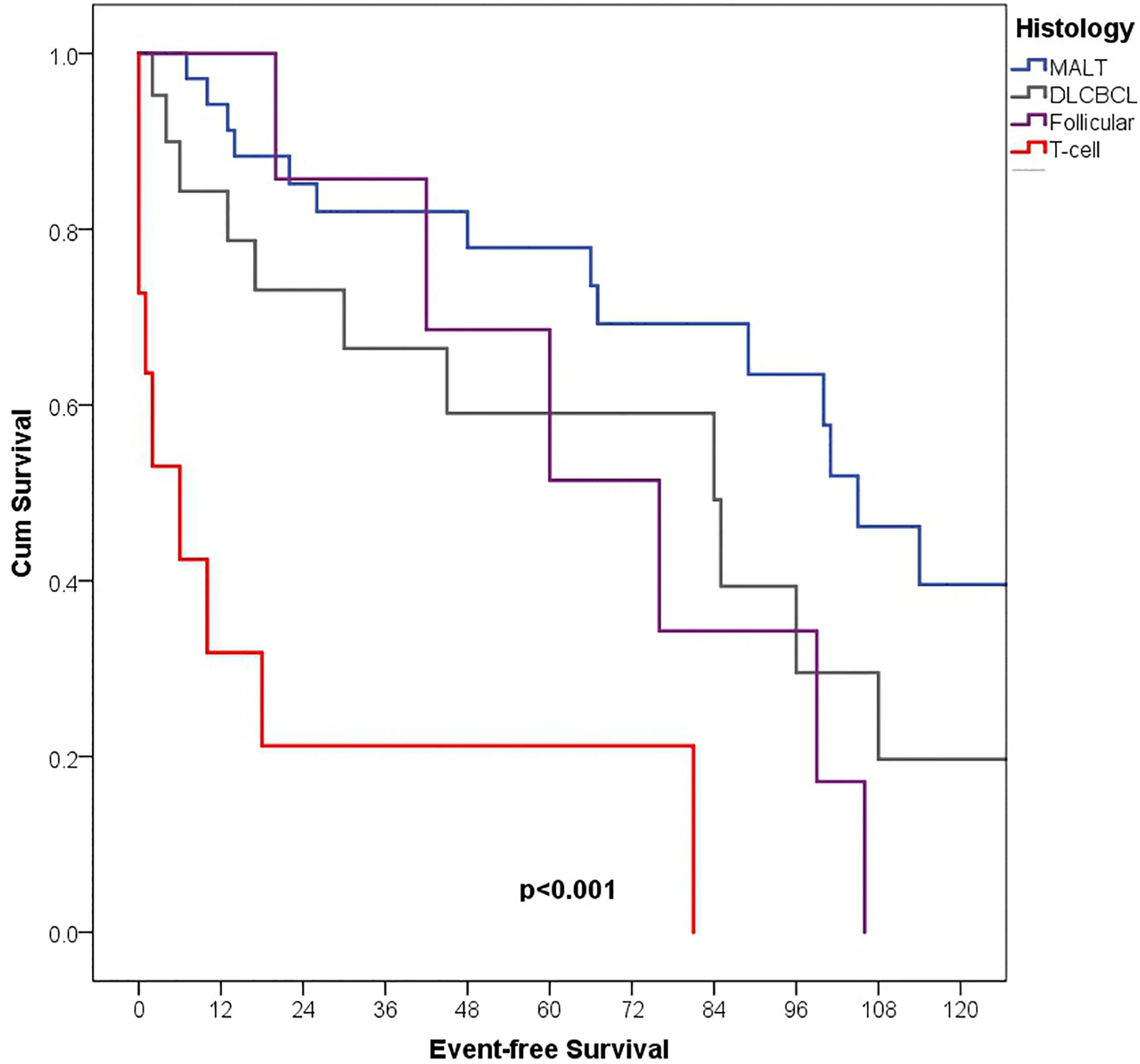
Kaplan–Meier overall survival curve of Hispanic patients with ocular-adnexal non-Hodgkin lymphoma stratified by tumor histology.

**Fig 5:**
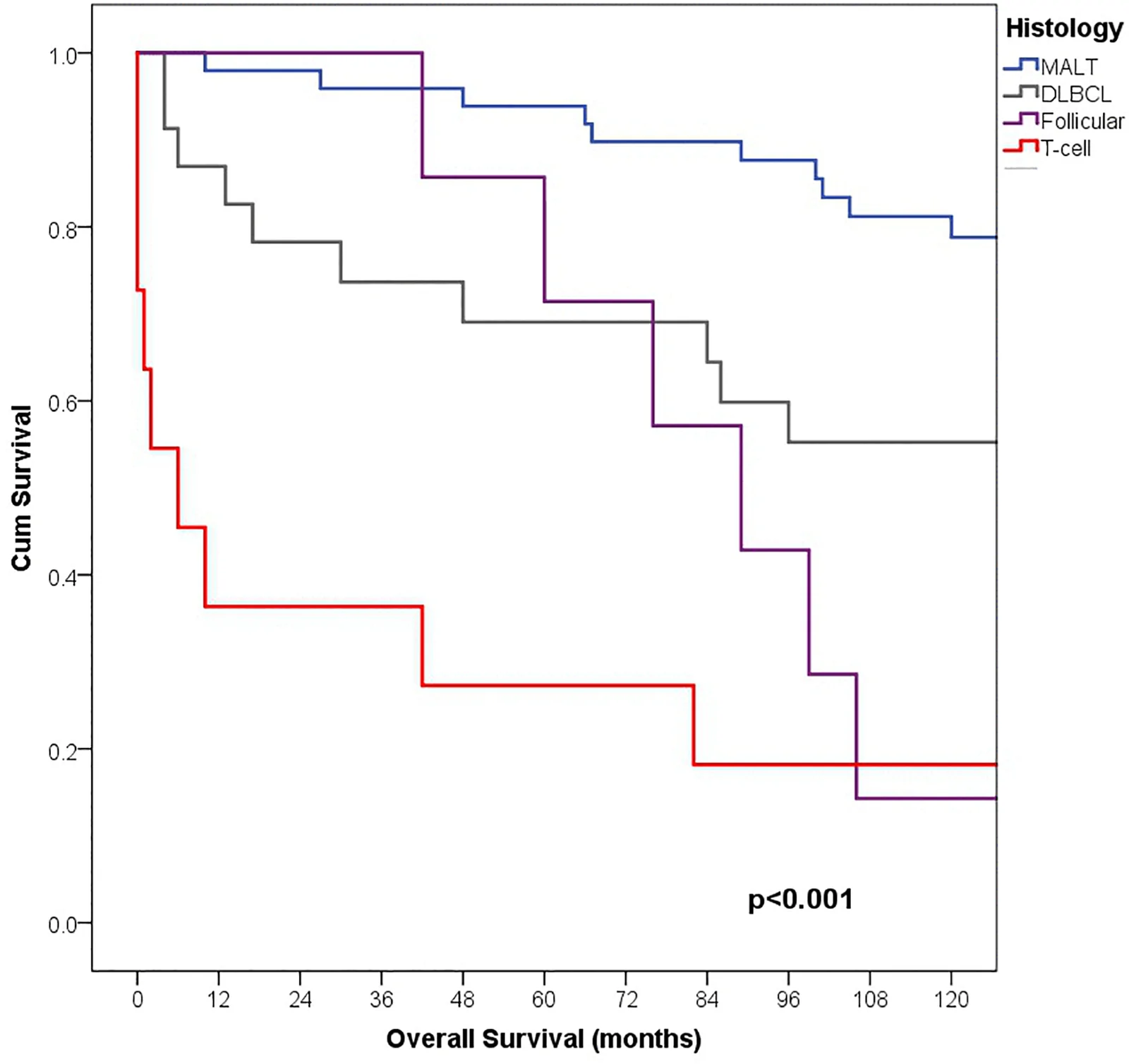
Kaplan–Meier event-free survival curve of Hispanic patients with ocular-adnexal non-Hodgkin lymphoma stratified by tumor histology.

Survival also differed according to lymphoma lineage and histologic subtype. Patients with B-cell lymphoma experienced superior survival compared with those with T-cell lymphoma (5-year OS: 87.1% vs. 33.3%; 5-year EFS: 69.7% vs. 22.2%; both p < 0.01) (Figs 4 and 5). Among B-cell lymphomas, MALT lymphoma demonstrated the most favorable outcomes, followed by follicular lymphoma and diffuse large B-cell lymphoma. Five-year overall survival was 93.9%, 85.7%, and 69.1%, respectively, whereas five-year event-free survival showed more heterogeneous outcomes across subtypes, 77.9%, 51.4%, and 59.1%, respectively.

For the cohort as a whole, median follow-up was 20.2 years. On multivariable analysis, increasing age remained an independent predictor of inferior disease-free survival (HR 1.084, 95% CI 1.028–1.142; p = 0.003) and overall survival (HR 1.092, 95% CI 1.037–1.150; p = 0.001) among patients with indolent lymphoma. Sex, ECOG performance status, clinical stage, and treatment modality were not independently associated with disease-free or overall survival in this subgroup. (Tables 2 and 3).

**Table 2:** Univariate and multivariate analyses of prognostic factors for disease-free survival in Hispanic patients with indolent ocular-adnexal non-Hodgkin lymphoma.

Characteristics	Disease-free Survival					
	Univariate			Multivariable		
	HR	95%CI	P value	HR	95%CI	P value
Age	1.052	1.015-1.090	0.005	1.084	1.028-1.142	0.003
Sex						
Female	1.000			1.000		
Male	0.874	0.372-2.056	0.874	1.782	0.590-5.385	0.305
ECOG						
0-1	1.000			1.000		
2-4	0.844	0.191-3.736	0.823	0.759	0.076-7.547	0.759
Clinical Stage						
I	1.000			1.000		
II	0.728	0.246-2.152	0.566	1.437	0.309-6.672	0.643
Treatment						
None	1.000			1.000		
RT	0.457	0.174-1.202	0.112	1.328	0.383-4.609	0.655
CT	1.270	0.439-3.671	0.659	3.644	0.943-9.085	0.061

Eastern Cooperative Oncology Group performance status; RT, Radiotherapy; CT, chemotherapy

**Table 3:** Univariate and multivariate analyses of prognostic factors for overall survival in Hispanic patients with indolent ocular-adnexal non-Hodgkin lymphoma.

Characteristics	Overall Survival					
	Univariate			Multivariable		
	HR	95%CI	P value	HR	95%CI	P value
Age	1.078	1.037-1.121	<0.001	1.092	1.037-1.150	0.001
Sex						
Female	1.000			1.000		
Male	0.912	0.372-2.236	0.841	0.898	0.351-2.302	0.898
ECOG						
0-1	1.000			1.000		
2-4	4.686	1.357-9.185	0.015	2.615	0.397-9.209	0.317
Clinical Stage						
I	1.000			1.000		
II	1.385	0.502-3.818	0.529	1.915	0.414-8.850	0.405
Treatment						
None	1.000			1.000		
RT	0.686	0.250-1.883	0.465	0.729	0.238-2.233	0.580
CT	1.101	0.338-3.583	0.873	1.961	0.498-7.724	0.335

Eastern Cooperative Oncology Group performance status; RT, Radiotherapy; CT, chemotherapy

Among patients with aggressive lymphoma, treatment modality was the main independent prognostic factor for disease-free survival. Compared with observation alone, combined chemoradiotherapy (HR 0.112, 95% CI 0.023–0.547; p = 0.007) and chemotherapy alone (HR 0.135, 95% CI 0.031–0.595; p = 0.008) were independently associated with improved disease-free survival. Radiotherapy alone demonstrated a nonsignificant trend toward improved disease-free survival (HR 0.200, 95% CI 0.033–1.203; p = 0.079). No clinicopathologic variable, including age, sex, ECOG performance status, clinical stage, or treatment modality, was independently associated with overall survival in patients with aggressive lymphoma (Tables 4 and 5).

**Table 4:** Univariate and multivariate analyses of prognostic factors for disease-free survival in Hispanic patients with aggressive ocular-adnexal non-Hodgkin lymphoma.

Characteristics	Disease-free Survival					
	Univariate			Multivariable		
	HR	95%CI	P value	HR	95%CI	P value
Age	1.008	0.986-1.031	0.463	0.997	0.972-1.023	0.834
Sex						
Female	1.000			1.000		
Male	0.801	0.336-1.909	0.617	0.461	0.142-1.493	0.196
ECOG						
0-1	1.000			1.000		
2-4	1.020	0.399-2.609	0.967	1.842	0.522-6.503	0.343
Clinical Stage						
I	1.000			1.000		
II	0.776	0.263-2.289	0.646	0.346	0.095-1.257	0.107
Treatment						
None	1.000			1.000		
RT	0.263	0.061-1.147	0.075	0.200	0.033-1.203	0.079
RT+CT	0.238	0.069-0.829	0.024	0.112	0.023-0.547	0.007
CT	0.256	0.087-0.756	0.014	0.135	0.031-0.595	0.008

Eastern Cooperative Oncology Group performance status; RT, Radiotherapy; CT, chemotherapy

**Table 5:** Univariate and multivariate analyses of prognostic factors for overall survival in Hispanic patients with aggressive ocular-adnexal non-Hodgkin lymphoma.

Characteristics	Overall Survival					
	Univariate			Multivariable		
	HR	95%CI	P value	HR	95%CI	P value
Age	1.023	0.999-1.047	0.060	1.006	0.982-1.031	0.601
Sex						
Female	1.000			1.000		
Male	0.480	0.196-1.178	0.109	0.318	0.094-1.072	0.065
ECOG						
0-1	1.000			1.000		
2-4	2.253	0.911-5.574	0.079	0.377	0.117-1.217	0.103
Clinical Stage						
I	1.000			1.000		
II	0.852	0.316-2.298	0.752	0.377	0.117-1.217	0.103
Treatment						
None	1.000			1.000		
RT	0.657	0.133-3.142	0.590	0.473	0.072-3.104	0.435
RT+CT	0.678	0.214-2.146	0.678	0.257	0.058-1.152	0.076
CT	0.496	0.184-1.339	0.496	0.222	0.059-1.834	0.126

Eastern Cooperative Oncology Group performance status; RT, Radiotherapy; CT, chemotherapy

## Discussion

In this single-center cohort of 98 patients with POANHL, older age was strongly associated with markedly inferior survival outcomes. Underlying lymphoma histologies vary widely from aggressive to indolent subtypes, highlighting the biologic heterogeneity of POANHL. In our cohort, MALT lymphoma was the most frequent histologic subtype (50.0%), followed by DLBCL and follicular lymphoma. This predominance of MALT-type ocular‑adnexal lymphoma aligns with prior large series reporting extranodal marginal zone lymphomas as the dominant subtype in primary ocular adnexal lymphoma, often representing the majority of cases, up to 75–80% or higher in Western cohorts, and reflecting the indolent nature of the disease compared with more aggressive subtypes such as DLBCL [22].

The age distribution was consistent with previously reported studies [1,14]. Prior studies describe a typical presentation between 45 and 70 years of age for indolent POANHL [14,23], comparable to the mean age observed in our cohort (58.0 ± 17.5 years). Although POANHL can occur at any age, including in younger adults, 19.4% of our cohort was under 45 years of age. This may be associated with regional etiologic factors such as *Chlamydia psittaci*, which has been implicated in the lymphomagenesis of MALT type POANHL in previous international studies [6,7]. Nevertheless, this finding should be interpreted cautiously because the present study was not designed to evaluate age-specific disease incidence. Larger population-based studies are needed to determine whether this observation reflects regional epidemiologic differences or normal variation within POANHL

POANHL in our cohort most commonly involved the orbit (42.8%), followed by the eyelid (29.6%) and conjunctiva (27.6%). Prior series similarly identify the orbit as the predominant site of disease [24,25], although some studies have reported conjunctival involvement as the most frequent presentation [4,26,27]. Bilateral involvement was uncommon in our cohort (8.2%), consistent with international reports indicating bilateral disease in fewer than 10% of cases [28,29]. The study cohort was restricted to patients with localized disease (Ann Arbor stage I–II), consistent with the typical presentation of POANHL, which commonly remains confined to the ocular adnexa at diagnosis [15,30].

In our cohort, poor performance status (ECOG 2–4) was significantly more prevalent among patients with aggressive lymphomas compared with those with indolent disease (20.0% vs 5.2%; p = 0.022). This distribution is consistent with prior reports indicating that higher-grade ocular adnexal lymphomas more often present with advanced or symptomatic disease and greater functional impairment compared with indolent subtypes, which frequently remain localized at diagnosis [11,31]. Although previous studies have identified ECOG performance status as an independent prognostic factor in POANHL [32], the close relationship between histologic aggressiveness and performance status in our cohort makes it difficult to fully distinguish the independent contribution of each variable to survival outcomes. Consequently, ECOG should be interpreted within the broader clinical context of disease biology and stage rather than as an isolated prognostic marker.

Treatment allocation in this cohort was determined by disease stage, histologic subtype, anatomic extent of disease, and treating physician judgment rather than by random assignment. Consequently, observed associations between treatment modality and survival should be interpreted as hypothesis-generating and reflective of real-world clinical practice rather than evidence of a causal treatment effect. This consideration is particularly important when comparing outcomes across treatment groups, as patients receiving combined chemoradiotherapy generally had more aggressive disease characteristics than those managed with radiotherapy alone or observation.

Our findings are consistent with contemporary therapeutic paradigms for ocular adnexal lymphoma. RT remains the standard first-line treatment for localized indolent primary ocular adnexal MALT lymphoma, with reported high response rates and durable local control across large cohorts and systematic reviews [1,17,33]. In our cohort, 24.5% of patients received RT. Among patients with aggressive lymphoma, chemotherapy alone (HR 0.135, p = 0.008) and combined chemoradiotherapy (HR 0.112, p = 0.007) were independently associated with improved disease-free survival compared with observation, whereas radiotherapy alone showed a nonsignificant trend (HR 0.200, p = 0.079). In contrast, no treatment modality was independently associated with disease-free or overall survival among patients with indolent lymphoma, or with overall survival among patients with aggressive lymphoma. However, treatment selection was strongly influenced by disease stage, histologic subtype, and physician judgment. Consequently, these findings should not be interpreted as evidence supporting routine combined chemoradiotherapy for all patients, particularly those with localized indolent disease, for whom radiotherapy alone remains an established standard of care. Given the retrospective design and non-random treatment allocation, these associations should be interpreted cautiously and viewed as hypothesis-generating rather than evidence of a causal treatment effect.

Although RT is highly effective for localized indolent disease, systemic therapy is crucial for aggressive histologic subtypes or bulky/advanced presentations. In our cohort, chemotherapy was used more frequently in aggressive lymphomas (66.7% vs 20.7%; p < 0.001), with CHOP‑based regimens being the most common. CR among patients receiving CHOP‑based therapy was 39.5%, consistent with prior reports in ocular‑adnexal DLBCL and other aggressive NHLs, where response rates range from 40–70% depending on disease stage, histology, and the addition of rituximab or other targeted agents [13,17,34,35]. These findings highlight that, while CHOP‑based therapy achieves meaningful responses, outcomes in aggressive POANHL remain inferior to indolent subtypes, underscoring the need for risk-adapted strategies, potential incorporation of novel agents, and individualized intensification when appropriate.

Overall, 45.6% of patients in our series achieved CR (53.8% for indolent vs. 34.5% for aggressive subtypes), with partial responses observed in 41.2%. These rates are in line with other institutional series of ocular‑adnexal lymphoma reporting high response with combined-modality therapy and relatively low rates of severe toxicity [13,35,36]. Treatment-related toxicities documented in the medical record were uncommon and were predominantly mild. Because adverse events were collected retrospectively, the incidence of treatment-related toxicity is likely underestimated and should be interpreted cautiously. Nevertheless, the observed variation in treatment approaches, together with the substantial proportion of patients managed without active therapy, highlights the need for additional prospective studies to better define evidence-based treatment strategies for POANHL.

On multivariable analysis, increasing age remained an independent adverse prognostic factor for both disease-free survival (HR 1.084; p = 0.003) and overall survival (HR 1.092; p = 0.001) among patients with indolent lymphoma, consistent with previously reported prognostic models identifying older age as a predictor of inferior survival in ocular adnexal lymphoma [3,12,13]. In contrast, among patients with aggressive lymphoma, treatment modality was the primary determinant of disease-free survival, with chemotherapy alone (HR 0.135; p = 0.008) and combined chemoradiotherapy (HR 0.112; p = 0.007) independently associated with improved disease-free survival compared with observation [17,33]. No clinicopathologic variable was independently associated with overall survival in the aggressive subgroup. Collectively, these findings support risk-adapted management based on patient characteristics and tumor biology while underscoring the need for further validation in larger multicenter cohorts [12,13,25].

The strengths of this study include its relatively large cohort, the largest reported in a Latin American tertiary cancer center, and an extended follow-up period. Limitations include the retrospective design, which carries an inherit risk of incomplete or inaccurately recorded clinical and pathological data, potentially affecting analytic precision. Although large within the regional context, the sample size remains modest compared with series from high income countries and may affect statistical power to detect subtle associations. In addition, the inclusion of both early (stage I–II) and a limited number of more advanced presentations introduces potential clinical heterogeneity; although analyses were focused on localized disease, residual confounding between histologic aggressiveness and disease extent may persist and should be interpreted cautiously. Furthermore, the interaction between histologic subtype, ECOG performance status, and outcomes may introduce residual confounding in multivariable models, and results should therefore be interpreted separately within indolent and aggressive subgroups. Changes in diagnostic approaches and treatment strategies over the study period may have introduced heterogeneity, complicating outcome comparisons over time. Patient-reported outcomes, including visual function and quality of life, were not captured. Treatment-related toxicity was collected retrospectively from medical records and may therefore be underestimated; therefore, safety conclusions should be limited to the low frequency of documented adverse events rather than definitive statements on tolerability. Treatment heterogeneity, in the absence of standardized national guidelines, may partly explain the less favorable long-term outcomes observed compared with reports from high-income countries. Additionally, the single-center, region-specific nature of this cohort may limit the generalizability of our findings to other populations or healthcare settings.

In conclusion, this study highlights the distinctive demographic and clinical profile of OANHL in a Hispanic–Latino population. Older age was independently associated with inferior survival among patients with indolent lymphoma, whereas treatment modality was independently associated with disease-free survival in aggressive lymphoma. However, these associations should be interpreted in the context of a retrospective, observational design and potential confounding by disease stage and histologic subtype. Because the study included patients with varying histologic subtypes and stage I-II disease, the observed association between histology and survival should be interpreted within the context of this heterogeneous population. Our findings are hypothesis-generating and may inform clinical decision-making in the absence of robust prospective clinical trial data.

## Supporting information

S1 File Deidentified Excel dataset containing clinical and pathologic information on Peruvian patients diagnosed with ocular-adnexal non-Hodgkin lymphoma. (XLSX)
