# Supplementary material for: Primary ocular-adnexal non-Hodgkin Lymphoma in a Peruvian cohort: An 18-year experience
Source: PLoS One. 2026 Aug 25;21(8):e0356381. doi: 10.1371/journal.pone.0356381 (PMC13505898; doi:10.1371/journal.pone.0356381)
Supplement: Supplementary file 1 [file pone.0356381.s003.docx]

**RESPONSE TO REVIEWERS**

**Reviewer #1**

Authors present a single centre series of treatment of primary ocular adnexal lymphoma, a rare presentation of low grade iNHL that a priori, requires a significant effort to gather real-world data of efficacy of current therapies and outcomes. This is a valuable effort and I commend the authors for this. Design: A retrospective study is appropriate for reporting rare variants of lymphoma, the statistical analysis plan is appropriate and well detailed.

**Comment #1**

However, there is no consensus definition of POANHL, I would suggest the authors to narrow their utilized definition in the methods. In this respect I believe the authors attempted to be inclusive with the different histologies, but the inclusion of aggressive B-cell and T-cell histology that are stage III (i.e. systemic lymphomas that happen to have ocular adnexal/orbital involvement) is not appropriate as masks the outcome of truly localized indolent adnexal lymphomas with is the spirit of the separate classification used. This introduces bias in the management and outcome and does not allow to draw conclusions about the outcomes of indolent PAONHL, which I think is the main objective here.

I would suggest these aggressive histologies are reported separately, can be within the same paper, with an approximation of the incidence/prevalence of ocular adnexal involvement in their population and clinical implications such as CNS relapses.

**Response**

We thank the reviewer for this insightful and constructive comment. In response, we have refined the definition of primary ocular adnexal non-Hodgkin lymphoma (POANHL) in the Methods section to clearly specify that eligible patients had histopathologically and immunophenotypically confirmed lymphoma originating within the ocular adnexal tissues (orbit, conjunctiva, eyelid, or lacrimal gland) at presentation, without evidence of disseminated disease (Ann Arbor stage III–IV). Accordingly, patients with stage III–IV disease were excluded from the study to avoid inclusion of systemic lymphomas with secondary ocular adnexal involvement.

Furthermore, to address the clinical heterogeneity highlighted by the reviewer, we restructured the analyses by evaluating indolent and aggressive/highly aggressive lymphomas separately. All analyses have been run again, reflected in our results. Baseline characteristics, treatment response, and multivariable survival analyses are now presented independently for each group, thereby minimizing bias arising from differences in tumor biology, treatment strategies, and prognosis. These changes allow a more appropriate evaluation of outcomes in localized indolent POANHL while still reporting the characteristics and outcomes of localized aggressive ocular adnexal lymphomas within the same study.

**Comment #2**

Reviewer comment:

I would urge the authors to be more broad in the evaluation of the importance of a cohort of PAONHL. The underrepresentation of Latin-American population is mentioned several times (may be too many). This is appropriate to highlight (reviewer is also South American), but you need to give your data collection its own value beyond the latin American population, I think the numbers are significant and there is scant literature of this. You should highlight the lack of data in the whole of the literature are your main justification for the work.

**Response:**

We appreciate this insightful recommendation and agree that the overall scarcity of high-quality real-world evidence represents the primary rationale for our study. Accordingly, we substantially revised the Introduction to broaden the scientific justification of the manuscript.

Specifically, we now emphasize that POANHL remains an uncommon malignancy with limited long-term outcome data worldwide and that most available evidence originates from relatively small retrospective institutional series. Given this context, our cohort provides one of the larger single-institution experiences with extended follow-up in the literature. The importance of our cohort is now presented primarily as a contribution to the global literature on POANHL, while the inclusion of a Hispanic-Latino population is described as an additional strength rather than the principal justification for the study. Redundant references to the Latin American population throughout the Introduction were removed or consolidated to improve readability.

**Comment #3**

Reviewer comment:

Probably not necessary to include that many details of the size and characteristics of your centre, suffice to say it is a national reference centred for haematological malignancies in Peru.

Response:

We agree with the reviewer’s suggestion and have simplified the description of the study setting in the Methods section. The previous detailed information regarding institutional size and annual clinical volume was removed and replaced with a concise statement identifying the Instituto Nacional de Enfermedades Neoplásicas (INEN) as Peru’s national tertiary referral center for oncology and hematologic malignancies. This modification improves clarity and readability while preserving essential contextual information.

**Comment #4**

Reviewer comment:

The differences in treatment modality between aggressive and indolent histologies, highlight my previous comments about the inclusion of these in the cohort and introducing significant bias when you are assessing outcomes such as response rate/PFS/OS combining both in the analyses. I would suggest presenting outcome separately for indolent and aggressive disease (ensuring state III-IV are excluded), including MVA. Presenting results in this way will allow readers to interpret findings easier and provide more valuable clinically-applicable information.

**Response:**

We appreciate this important and insightful recommendation. In response, we substantially revised the Results section to address concerns regarding potential bias introduced by pooling biologically and clinically distinct disease entities, and to improve the interpretability of outcomes by histologic subtype.

Specifically, we:

- Reorganized the cohort definition and confirmed that patients with Ann Arbor stage III–IV disease were excluded to avoid inclusion of systemic lymphomas with secondary ocular adnexal involvement.
- Reorganized the Treatment Patterns section to describe management stratified by disease stage and histologic subtype prior to presenting overall treatment distributions.
- Reorganized the Treatment Response section to report outcomes separately for patients with indolent and aggressive/highly aggressive lymphoma.
- Reorganized the Survival section to present Kaplan Meier analyses stratified by histologic subtype.
- Performed and reported multivariable Cox proportional hazards models separately for indolent and aggressive/highly aggressive lymphoma cohorts, allowing independent assessment of prognostic factors within each biologically distinct subgroup.

These revisions directly address the concern regarding heterogeneity and potential bias in pooled analyses and improve the clinical interpretability and applicability of the findings.

**Comment #5**

Reviewer comment:

To claim there is a predominance in young patients of PAONHL locally you need to provide evidence of PAONHL indigence in younger patients in other contexts, or else, provide local evidence of incidence of other lymphomas under 45 years that is significantly lower than 8,5%.

The conclusion about performance status and outcomes, needs to be drawn separately for indolent and aggressive conditions. There is a significant interaction between histology, performance status and outcomes, so in a MVA this will lead to bias.

Your conclusion suggests that all patients should have CRT. This needs to be expanded trying to identify factors that would help the reader identity patient who would benefit more from CRT as opposed to RT, particularly for those who are true stage I

I disagree with the conclusion drawn in the 6th paragraph of the discussion, a retrospective review with likely incomplete recollection of adverse events will not allow such conclusion to be made, also don’t see a link between safety and heterogeneity of population.. rephrase this to reflect the toxicity observed was low without going further.

The aggressive histology as a factor associated with outcomes needs to be confirmed without the cases with stage III disease.

Praise the limitations paragraph.

**Response:**

We thank the reviewer for these thoughtful recommendations. We have carefully revised the Discussion and Conclusions to address each of the concerns raised and to provide a more balanced interpretation of our findings.

Specifically:

- The discussion regarding younger patients was revised to correct the proportion of patients younger than 45 years from the previously reported value to the accurate figure (19.4%). In addition, we removed any implication of a higher regional prevalence of POANHL among younger individuals. The revised text now presents this finding as a descriptive characteristic of our cohort without inferring age-specific incidence or comparisons with other lymphoma subtypes.
- The interpretation of ECOG performance status was revised to explicitly acknowledge the interaction between histologic aggressiveness, functional status, and outcomes. We now emphasize that ECOG performance status should be interpreted within the broader context of disease biology rather than as an isolated prognostic factor.
- The section discussing treatment modality was substantially revised to avoid overinterpretation of the observed association between chemoradiotherapy and improved outcomes. We now explicitly state that treatment allocation was influenced by disease stage, histologic subtype, and physician judgment, and that these findings should be considered hypothesis-generating rather than evidence supporting routine use of combined chemoradiotherapy, particularly in localized indolent disease.
- The toxicity section was revised to remove any implication of definitive safety conclusions. We now report that treatment-related adverse events were infrequent but likely underestimated due to the retrospective nature of data collection, and we avoid extending conclusions beyond observed toxicity rates.
- The conclusions regarding aggressive histology were revised to ensure consistency with the stratified analyses and exclusion of advanced stage disease. We now state that aggressive histology was associated with inferior outcomes within this heterogeneous cohort, while acknowledging that outcomes must be interpreted in the context of disease stage and histologic subtype.

Collectively, these revisions substantially improve the balance and clinical interpretability of the Discussion and ensure that conclusions are fully aligned with the retrospective and observational nature of the study.

**Reviewer #2**

**Comment #1**

Reviewer comment:

IPI has described for all lymphoma subtypes - please could the authors detail if the score used varied based on the histological subtype?

**Response:**

Thank you for identifying this area requiring clarification. We expanded the Methods section to describe how the International Prognostic Index (IPI) was determined within our cohort.

The revised manuscript now specifies that the conventional IPI scoring system was applied uniformly across all lymphoma histologic subtypes, without modification according to histology, to allow standardized comparison of prognostic risk between patients. This approach was used to maintain consistency across a heterogeneous cohort including both indolent and aggressive lymphomas.

**Comment #2**

Reviewer comment:

The description of treatment patterns can be improved by describing the treatment of localised/stage 1 disease for indolent vs aggressive histology followed by treatment of stage II/III disease indolent vs aggressive.

**Response:**

We appreciate this excellent suggestion. In response, we have substantially reorganized the Treatment Patterns section to improve clarity of stage- and histology-specific management.

Specifically, the revised manuscript first describes treatment patterns in localized stage I disease stratified by histologic subtype (indolent vs aggressive), followed by stage II disease using the same stratified approach. As patients with Ann Arbor stage III–IV disease were excluded in accordance with prior methodological clarification to avoid inclusion of systemic lymphomas with secondary ocular adnexal involvement, treatment patterns for advanced-stage disease are not included.

Finally, overall treatment distributions are presented to provide a global summary of therapeutic approaches across the cohort.

This revised structure aligns treatment reporting with the final study design and improves interpretability of clinical decision-making across disease stages and histologic subtypes.
